# Superinfection of a Dead Hepatic Echinococcal Cyst with a Cutaneous Fistulization

**DOI:** 10.1155/2017/9393462

**Published:** 2017-10-18

**Authors:** Giuseppe Cicero, Alfredo Blandino, Giorgio Ascenti, Tommaso D'Angelo, Luciano Frosina, Carmela Visalli, Ignazio Salamone, Maria Adele Marino, Marco Cavallaro, Silvio Mazziotti

**Affiliations:** ^1^Section of Radiological Sciences, Department of Biomedical Sciences and Morphological and Functional Imaging, University of Messina, Policlinico “G. Martino”, Via Consolare Valeria 1, 98100 Messina, Italy; ^2^Department of Diagnostic and Interventional Radiology, Goethe University Hospital, Frankfurt, Germany

## Abstract

Cystic echinococcosis (CE), also known as “hydatid disease” (HD), is a zoonotic infection caused by the larval stage of* Echinococcus granulosus*, which infects humans as intermediate hosts through the orofecal route. Carried by the intestinal venous blood, the embryos released by the eggs of the tapeworms can reach every organ, especially the liver, turning into a hydatid cyst. Usually asymptomatic, the cysts can be incidentally detected through radiological examinations performed for other reasons. We show an unusual case of superinfection of a hydatid cyst with typical radiological features of inactivity (WHO-type CE5) with an even rarer skin fistulization passing through a subcutaneous-abdominal abscess involving the right iliac muscle.

## 1. Introduction

Cystic echinococcosis (CE), also known as “hydatid disease” (HD), is a zoonotic infection caused by the larval stage of* Echinococcus granulosus*, which accidentally infects humans through the orofecal route.

Although previously endemic in Africa, South America, and Eurasia, the disease is nowadays worldwide spread due to the increased migratory flows.

Because of the intestinal absorption, the main organ affected is the liver (70% of cases), where the hydatid cyst can develop.

In most cases, the disease is asymptomatic and the typical cystic lesion can be depicted as an incidentaloma while performing imaging examinations.

Otherwise symptoms may be aspecific (weight loss, anemia, fatigue, etc.) or related to complications, such as rupture of the cysts (spontaneous, traumatic, or iatrogenic), secondary infection, and cholangitis.

The final diagnosis is reached matching patient's clinical history, specific serologic tests, and imaging evaluation, helpful in providing a complete clinical picture [1].

According to the radiological findings, several classifications have been proposed, all in agreement in defining thick-calcified-wall cysts as inactive or dead.

We show an unusual case of superinfection of a dead calcified hydatid cyst (WHO-type CE5) [2] with an even rarer skin fistulization passing through a subcutaneous-abdominal abscess involving the right iliac muscle.

## 2. Case Report

A 68-year-old male patient suffering from chronic renal and heart failure and alcohol-related cirrhosis was admitted to our hospital with fever, abdominal pain, and a right-flank fistula, draining a huge quantity of purulent secretion.

**Figure 1:**
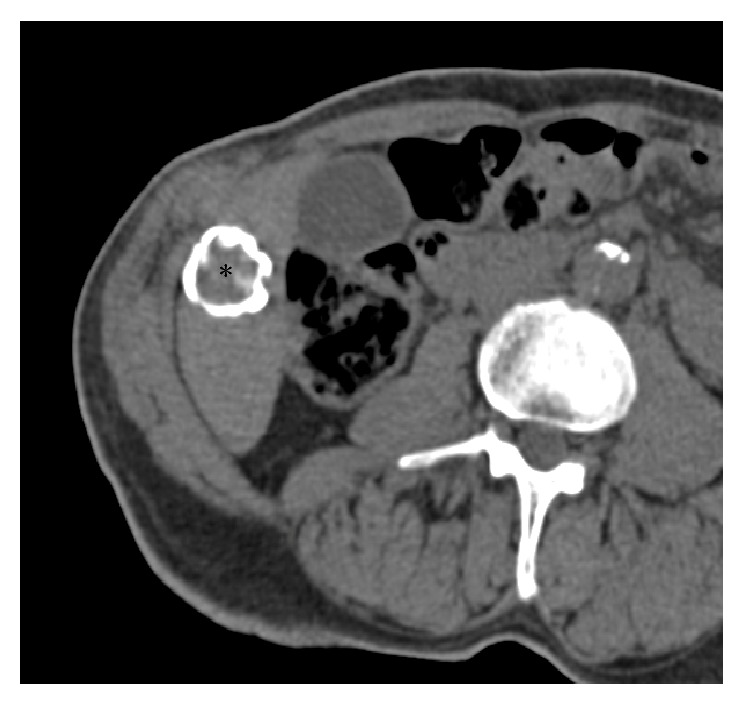
Axial unenhanced CT-scan performed a few years before the superinfection shows the hepatic hydatid cyst* (asterisk)* with calcified wall in segment VI.

He had also a known history of CE with a calcified cysts of the liver, incidentally discovered a few years before at a chest X-ray in our department and confirmed at unenhanced CT-scan (Figure 1).

Laboratory tests showed a neutrophilic leukocytosis (WBC 11300; *N*: 84%) and an electrolyte imbalance with severe hyponatremia.

An ultrasound (US) examination of the abdomen was immediately performed, showing the presence of a subcutaneous abscess.

To better evaluate the size and depth of the abscess he underwent an MR examination performed in our department using a 1.5 T MR Philips Gyroscan Intera (Philips Medical System, Best, Netherlands) and phased-array abdominal coils. Different pulse sequences were applied: 2D axial and coronal T2-weighted turbo spin echo (TSE) sequences, 2D axial echo-planar imaging (EPI) sequence at different *b* values (*b*: 0, 500, 800 s/mm^2^), and 2D T1-weighted axial dual fast-field-echo (FFE) breath-hold sequence.

Intravenous injection of contrast medium was avoided due to the chronic renal failure of the patient.

**Figure 2:**
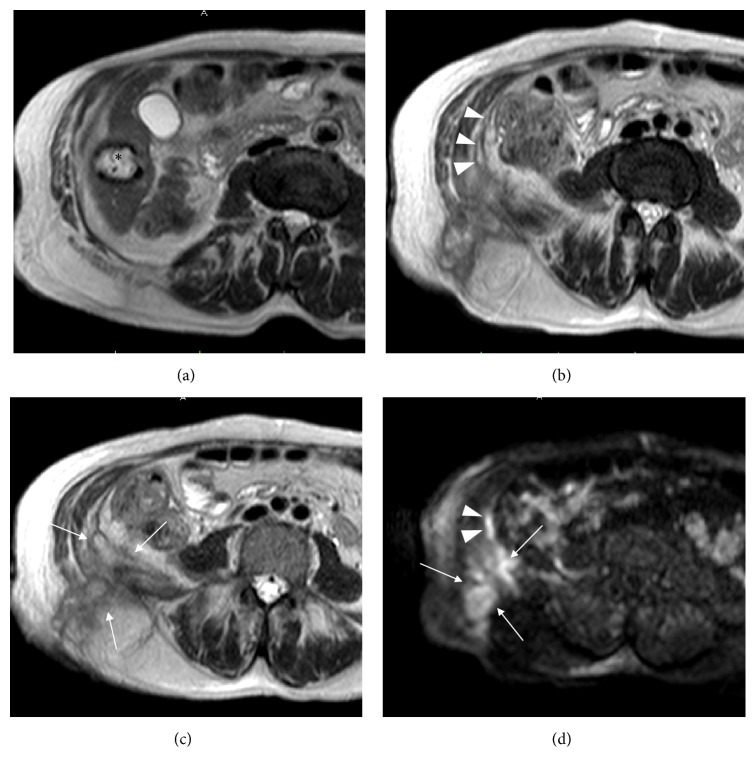
Axial MR TSE T2-weighted scans (a–c) show the hydatid cyst* (asterisk)*, with a soft hyperintensity of the surrounding hepatic parenchyma due to inflammation, the fistulous tract* (arrowheads)*, and the abdominal-subcutaneous abscess* (arrows)* involving the right iliac muscle. Axial diffusion weighted image (*b-*value 800 s/mm^2^) (d) demonstrates the high hyperintensity of both the abscess* (arrows)* and the fistula* (arrowheads)*.

MRI confirmed the presence of an inhomogeneous fluid collection with irregular peripheral walls, indicative of abscess, extending from the subcutaneous tissues of the posterior right abdominal flank into the abdominal cavity, through the right iliac muscle. The abscess showed a middle-low T2 hyperintensity of the content while DWI study revealed diffusion restriction of the lesion (Figure 2).

A long narrow fistula connecting the abscess with the hepatic hydatid cyst was also found.

**Figure 3:**
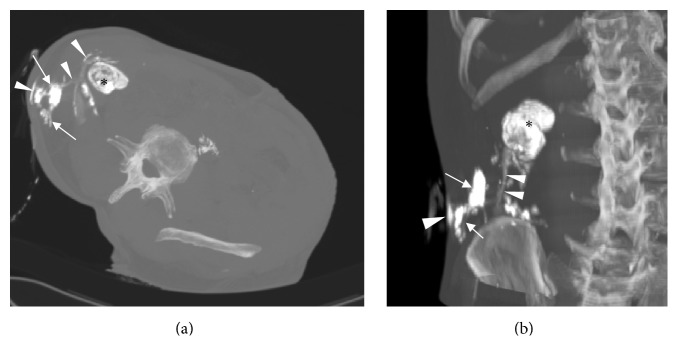
Axial oblique (a) and coronal (b) MIP reformatted CT-fistulography. The injection of the contrast agent through the external opening of the fistula allowed identifying the abscess* (arrows)* and the whole fistulous tract* (arrowheads)* ascending to the hepatic hydatid cyst* (black asterisk)*.

In order to better assess the route of that fistulous tract, a CT-fistulography was obtained catheterizing the external opening of the fistula with a thin cannula and injecting a water-soluble iodine contrast medium. The exam showed a progressive contrast filling of the abscess and the abdominal fistula (Figure 3), ascending till the calcified hydatid cyst of the liver.

The drainage culture test showed the presence of* Pseudomonas aeruginosa* and* Klebsiella oxytoca*, without any* Echinococcus*.

Afterwards, the patient underwent an antibiotic therapy with percutaneous drainage of the cutaneous-abdominal abscess. After the complete resolution of the abscess, a surgical cystectomy was performed.

## 3. Discussion

*Echinococcosis* is a worldwide zoonosis, caused by Cestode parasites, commonly known as small tapeworms of carnivorous animals [3], that can infect humans, as intermediate hosts, through the orofecal route.

The liver is the most common organ involved (75% cases of HD), followed by the lungs (15%) [4].

Although the course of the liver hydatid disease is usually asymptomatic, complicated forms are not rare, occurring in 30–60% of the patients [5].

The main complications include traumatic or idiopathic rupture of the cysts into the biliary tract, which is the most frequent, or into peritoneum, skin, digestive tract, or thorax, due to a transdiaphragmatic involvement [5, 6].

Large and superficial hepatic cysts are considered to be the most susceptible to break.

“Mass-effect” of large lesions may also cause vascular complications, such as Budd-Chiari and vena-cava syndromes, and biliary obstruction that may lead to cholestatic jaundice, cholangitis, biliary colic, and fever [7].

Suppuration of the cyst is caused by a cystobiliary communication and it is not a rare complication, with an occurrence of 5–40% [8].

However, although the association between calcified hydatid cysts and suppuration is well known [9], to our knowledge, there are not a clear percentage of occurrence and no imaging descriptions in cysts with an egg-shell thick calcified wall that is usually considered a feature of inactivity.

Certainly, imaging techniques play a pivotal role in a comprehensive evaluation of hydatid disease.

Though abdominal ultrasonography is considered the gold standard in identifying and characterizing the cysts, CT and MRI have reached an increasing importance over the years.

In fact, while CT-scan has a good sensitivity and specificity in the evaluation of hepatic HD, especially in depicting wall calcifications, MRI is nowadays considered the best imaging investigation in differentiating the fluid content of the cyst from other components and in depicting vascular or biliary tree involvement and extrahepatic complications [10, 11].

On the basis of imaging findings, several classifications have been proposed in typifying echinococcal cysts, but only few of them achieved a large consensus.

Through a sonographic evaluation, Gharbi et al. [12] proposed a subdivision of the hydatid cysts into five types: a simple fluid collection (type I), a fluid collection with split wall (type II), a fluid collection with septa (type III), a cyst with heterogeneous echo patterns (type IV), and a cyst with reflecting thick walls (type V).

In order to establish a simpler and standardised classification, also able to reflect the stages of the disease and the related treatment, a new one was introduced in 2003 by the Informal Working Group on Echinococcosis (IWGE-WHO). Still relayed on ultrasound examination, this classification recognizes six categories of hydatid cysts: CL, a simple cyst with anechoic content and not clearly visible wall, suspicious for an early stage of EC; CE1, a cyst with visible wall containing an inhomogeneous fluid due to the presence of hydatid sand; CE2, a multiseptate cyst with daughter cysts inside, with variable appearance (“rosetta-like,” “wheel-like,” or “honeycomb-like” structure); CE3, characterized by anechoic content with detached membranes within (3a) or daughter vesicle inside solid-echoic areas (3b), related to degenerated daughter cysts; CE4, hypoechoic or inhomogeneous content without daughter cysts; CE5, with thick calcified wall with a cone shadow. Moreover, these categories were grouped on the basis of their physiopathological behaviour into three types: active (CL, CE1, CE2), transitional (CE3), and inactive (CE4, CE5) [2].

However, the inclusion of CE4 type in the inactive group has raised some doubts, due to the presence of fertile liquid inside the vesicles; this implies a consequent “watch and wait” clinical approach [13].

Another widely used brief classification, suggested by Precetti et al. [14], divides the cysts in four types on the basis of the imaging findings: simple cyst with noninternal architecture (type I); cyst with daughter cysts and matrix (type II); calcified cyst (type III); complicated cyst (type IV).

Gharbi's, IWGE-WHO, and Precetti's categorizes are summarized and compared in Table 1.

Apart from Gharbi's one, which merely consists in a morphological description, the other two classifications consider cysts with a peripheral thick calcified wall as inactive and therefore less likely to undergo changes or complications [2, 8, 14].

Nowadays, three different options are available while treating uncomplicated CE: surgery, PAIR, and chemotherapy. CE4 and CE5 types are generally excluded from any kind of therapy and a “watch and wait” strategy is actually recommended.

Surgical treatment consists of different approaches, from the conservative (simple-closure tube drainage or marsupialization) to the radical ones (cystectomy or hepatic resections). Despite its invasiveness, it still remains the first choice in the treatment of large CE2–CE3b or complicated EC [8].

PAIR (an acronym that stands for “puncture, aspiration, injection, reaspiration”) technique has gained momentum during the last two decades, due to its lower invasiveness [15]. It consists in a US-guided needle aspiration of half volume of an EC followed by the injection of a hypertonic saline solution or ethanol [16] and it is mainly indicated for CE1 and CE3a cysts bigger than 5 cm. The main limitations of PAIR include biliary communication, infection of the cyst cavity, and, although very rare, anaphylactic reactions [17].

On the other hand, chemotherapy consists in the administration of mebendazole (MBZ) or albendazole (ABZ) and it can be combined as an adjuvant to surgery or PAIR [8, 17].

The treatment of CE2 and CE3b cysts has been discussed over the years, due to their typical trend to relapse. Although some works suggest to choose an expectant management, in particular for C3b types [18], Akhan et al. [19] have recently achieved better results in terms of decreased complications and lower recurrence, using a “modified catheterization” PAIR technique that also includes the removal of the solid components of the cyst.

As a matter of fact, in our case, the patient was affected by a hepatic hydatid cyst, previously demonstrated in a CT-scan performed in our department, completely surrounded by a thickened calcified wall.

Nevertheless, at the patient's latest hospitalization, a very serious superinfection of that cyst was found. In particular, sonography and MRI showed the presence of a large abscessual collection located in the subcutaneous tissues of the posterior right flank and extending into the abdominal cavity through the right iliac muscle.

Moreover, the abscess was in communication superiorly with the hydatid cyst of the liver, through a thin intra-abdominal fistula and inferolaterally to the skin surface.

In order to completely assess the length, the size, and the orientation of that fistula, a CT- fistulography was obtained, which clearly enhanced the whole route.

A cutaneous involvement with a spontaneous skin fistulization is considered a very rare complication of liver hydatidosis and only a few cases have been reported [20, 21].

Skin fistulization generally occurs at the same anatomic level of the hepatic cyst, as a result of some pathophysiological steps: protrusion of the cyst into the innermost muscular layer of the abdominal wall, penetration into the muscular tissue, subcutaneous rupture, and/or skin fistula formation [20].

In our case, a progressive cystic adhesion to the abdominal wall did not occur and the external opening was very far from the hepatic hydatid cyst, considering that the fistula was located in the lateral abdominal cavity and connected downwards to the subcutaneous-abdominal abscess which involved the right iliac muscle.

To our knowledge, the involvement of the iliac muscle has never been reported in the literature up to now.

In conclusion, we showed an unusual case of superinfection of an inactive calcified hydatid cyst with an even rarer abdominal-cutaneous fistulization passing through a subcutaneous-abdominal abscess involving the right iliac muscle.

Furthermore, it demonstrates the usefulness of MRI not only in identifying the cystic content, the extension of the disease, and the related complications but also in identifying the thin fistulous tract in order to allow a better treatment planning.

## Figures and Tables

**Table 1:** Comparison among Gharbi's, WHO, and Precetti's classifications of the echinococcal cysts.

Gharbi et al.	WHO	Precetti et al.

	CL	I
I	CE1	II
II	CE3A	
III	CE2–CE3B	
IV	CE4	
V	CE5	III
		IV

## References

[1] Pakala T., Molina M., Wu G. Y. (2016). Hepatic Echinococcal Cysts: A Review. *Journal of Clinical and Translational Hepatology*.

[2] WHO Informal Working Group (2003). International classification of ultrasound images in cystic echinococcosis for application in clinical and field epidemiological settings. *Acta Tropica*.

[3] Gottstein B. (2010). Echinococcus spp. and echinococcosis. *Acta Veterinaria Scandinavica*.

[4] Beggs I. (1985). The radiology of hydatid disease. *American Journal of Roentgenology*.

[5] Symeonidis N., Pavlidis T., Baltatzis M. (2013). Complicated liver echinococcosis: 30 years of experience from an endemic area. *Scandinavian Journal of Surgery*.

[6] Dziri C., Haouet K., Fingerhut A., Zaouche A. (2009). Management of cystic echinococcosis complications and dissemination: Where is the evidence?. *World Journal of Surgery*.

[7] Rinaldi F., Brunetti E., Neumayr A., Maestri M., Goblirsch S., Tamarozzi F. (2014). Cystic echinococcosis of the liver: A primer for hepatologists. *World Journal of Hepatology*.

[8] Nunnari G., Pinzone M. R., Gruttadauria S. (2012). Hepatic echinococcosis: clinical and therapeutic aspects. *World Journal of Gastroenterology*.

[9] Prousalidis J., Tzardinoglou E., Kosmidis C., Katsohis K., Aletras O. (1999). Surgical management of calcified hydatid cysts of the liver. *HPB Surgery*.

[10] Marrone G., Crino F., Caruso S. (2012). Multidisciplinary imaging of liver hydatidosis. *World Journal of Gastroenterology*.

[11] Mazziotti S., Gaeta M., Blandino A., Barone M., Salamone I. (2000). Hepatobronchial fistula due to transphrenic migration of hepatic echinococcosis: MR demonstration. *Abdominal Imaging*.

[12] Gharbi H. A., Hassine W., Brauner M. W., Dupuch K. (1981). Ultrasound examination of the hydatic liver. *Radiology*.

[13] Da Silva A. M. (2010). Human echinococcosis: A neglected disease. *Gastroenterology Research and Practice*.

[14] Precetti S., Gandon Y., Vilgrain V. (2007). Imaging of cystic liver diseases. *Journal de Radiologie*.

[15] Akhan O., Özmen M. N., Dinçer A., Sayek I., Göçmen A. (1996). Liver hydatid disease: long-term results of percutaneous treatment. *Radiology*.

[16] Üstünsöz B., Akhan O., Kamiloğlu M. A., Somuncu I., Uğurel M. Ş., Çetiner S. (1999). Percutaneous treatment of hydatid cysts of the liver: Long-term results. *American Journal of Roentgenology*.

[17] Tamarozzi F., Vuitton L., Brunetti E., Vuitton D. A., Koch S. (2014). Non-surgical and non-chemical attempts to treat echinococcosis: do they work?. *Parasite*.

[18] Rinaldi F., De Silvestri A., Tamarozzi F., Cattaneo F., Lissandrin R., Brunetti E. (2014). Medical treatment versus "Watch and Wait" in the clinical management of CE3b echinococcal cysts of the liver. *BMC Infectious Diseases*.

[19] Akhan O., Salik A. E., Ciftci T., Akinci D., Islim F., Akpinar B. (2017). Comparison of long-term results of percutaneous treatment techniques for hepatic cystic echinococcosis types 2 and 3b. *American Journal of Roentgenology*.

[20] Kjossev K. T., Teodosiev I. L. (2013). Cutaneous fistula of liver echinococcal cyst previously misdiagnosed as fistulizated rib osteomyelitis. *Tropical Parasitology*.

[21] Bahce Z. S., Akbulut S., Aday U., Demircan F., Senol A. (2016). Cutaneous fistulization of the hydatid disease A PRISMA-compliant systematic review. *Medicine (United States)*.

